# Bioinformatics Analysis of the Expression of ATP Binding Cassette Subfamily C Member 3 (ABCC3) in Human Glioma

**DOI:** 10.1515/med-2020-0016

**Published:** 2020-02-28

**Authors:** Zelin Sun, Xiaoyuan Qi, Yan Zhang

**Affiliations:** 1 Department of Clinical Laboratory, North China University of Science and Technology Affiliated Hospital, Tangshan Hebei Province 063000 PR China; 2 Department of Neurosurgery, North China University of Science and Technology Affiliated Hospital, Tangshan Hebei Province 063000 PR China; 3 Department of Family Planning ,Tangshan Municipal Maternal and Child Health Care Hospital, Tangshan Hebei Province 063000 PR China

**Keywords:** Glioma, *ABCC3* gene, Bioinformatics, TCGA database, Survival analysis

## Abstract

**Objective:**

To investigate the expression of the ABCC3 gene in human glioma and its correlation with the patient’s prognosis.

**Methods:**

The cancer genome atlas (TCGA) database was used to analyze the differential expression of the ABCC3 gene in human glioma. The STRING database was used to construct the protein-protein interaction (PPI) network of the ABCC3 gene coding protein. The co-expression genes relevant to the ABCC3 gene were analyzed by the Pearson correlation test. A log-rank test was used to analyze the difference of overall survival (OS) and disease-free survival (DFS) between the high and low ABCC3 gene expression groups.

**Results:**

The expression level of the ABCC3 gene in glioma tissues was lower than that of corresponding normal brain tissues. The PPI network contains 51 nodes with the average node degree of 13.3 and the local clustering coefficient of 0.72 which indicated that the PPI enrichment was significant (p<0.001). Ten hub genes (ABCC3,NR1I2,NR1H4,-CYP7A1,SLC10A1,CYP3A4,UGT1A1,UGT1A8,UGT1A6 and ALB) were identified by the cytoscape software. The KEGG analysis was enriched in drug metabolism - cytochrome P450 and PPAR signaling pathway. CFI gene expression level was positive correlated with the ABCC3 expression level (r=0.71, p<0.05). And the CNRIP1 gene expressed was negative correlated with ABCC3 expression (r=-0.43, p<0.05). The overall survival (HR=2.8, P<0.05) and disease-free survival rates (HR=2.0, P<0.05) of patients with ABCC3 low expression glioma were significantly higher than those of patients with high expression of ABCC3. Conclusion The expression level of the ABCC3 gene in glioma was decreased compared to normal brain tissue. The overall survival and disease-free survival of in the ABCC3 low-expression group was significant decreased.

## 1 Introduction

Glioma is a group of tumors in central nervous system. Glioma accounts for about 30% of all primary brain tumors, and 50% of gliomas are WHO IV glioblastoma with the most malignant degree [1, 2]. According to the clinical and histopathological characteristics, gliomas are generally divided into four grades (Grade I to IV). The prognosis of IV glioblastoma patients is extremely poor. Over 80% of patients have a total survival of less than 12 months and a long-term survival rate of less than 5% in grade IV glioblastoma [3, 4]. The main reason for poor prognosis of IV glioblastoma is related to malignant biological behavior of tumor cells, including rapid proliferation and marked aggressive growth [5].

It is known that ATP-binding cassette transporters (ABC transporters) regulate the traffic of multiple compounds, including chemotherapeutic agents, through biological membranes. ABC transporters are expressed in many kinds of tumor cells and correlated with tumor drug resistance. Despite many studies that have investigated ABC transporters in tumor drug resistance and its mechanisms, little is known about their expression and clinical value in glioblastoma (GBM). Dréan and his colleagues evaluated the ATP binding cassette transporters: their expression and clinical values in glioblastoma, and found that expression of ABC transporters was detected in GBM and microenvironmental cells and better reproduced in GBM-PDCL. ABCA13 expression is an independent prognostic factor in newly diagnosed GBM patients[6]. ATP binding cassette subfamily C member 3 (ABCC3) also known as ABC31, EST90757, MLP2, MOAT-D, MRP3, cMOAT2 located in chromosome 17: 50,634,777-50,692,252 of a human being. The protein encoded by *ABCC3* is a member of the superfamily of the ATP-binding cassette (ABC) transporters [7]. The specific function of this protein has not yet been determined.

## 2 Material and methods

### 2.1 Expression of ABCC3 gene in glioma

In the TCGA database, “glioma” and “ABCC3 6 gene” were searched for in humans. The expression level of *ABCC3* gene in glioma and corresponding normal tissues was analyzed and compared. At the same time, the expression level of the *ABCC3* gene in various human tumors was analyzed.

### 2.2 GO and KEGG enrichment

Functional enrichment and signal pathway analysis of *ABCC3* genes were analyzed by gene ontology (GO) and Kyoto Encyclopedia of Genes and Genomes (KEGG) [8, 9]. Gene ontology (GO) [10, 11] includes biological process (BP), cellular component (CC) and molecular function (MF).

### 2.3 PPI network analysis and hub gene identification

The PPI network of the ABCC3 gene was constructed by STRING (http://string-db.org/cgi/input.pl) [12]. Cytoscape software was used to screen the key genes in protein network.

### 2.4 Survival analysis

According to the median expression level of *ABCC3* in glioma tissues, the patients were divided into high and low expression groups. Survival analysis (Kaplan-Meier curve) and log-rank test were used to calculate overall survival (OS) and disease-free survival (DFS) of the high and low expression groups. P<0.05 was considered significant statistical difference.

## 3 Result

### 3.1 *ABCC3* expression in Glioma

**Figure 1:**
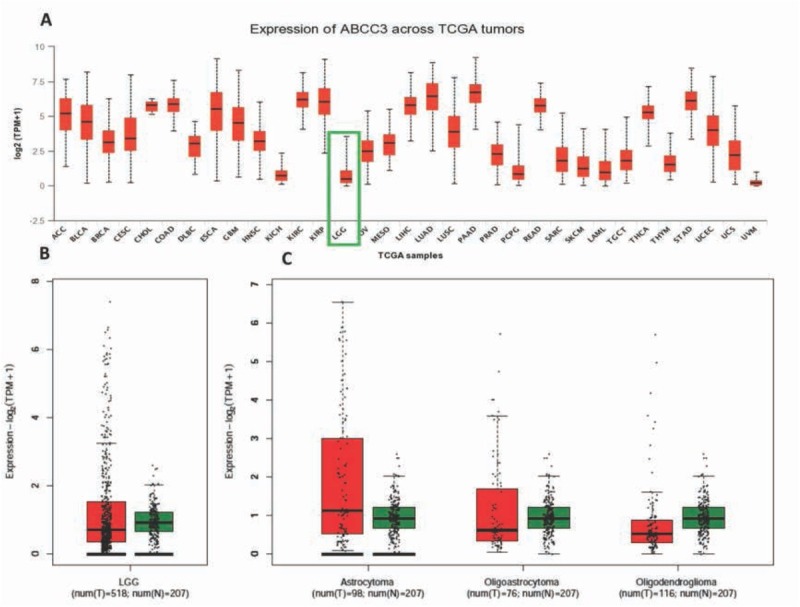
The expression of ABCC3 in human glioma tissues and other tumors (A: ABCC3 gene expression in various solid tumors and corresponding normal tissues; B: Scatter plot of ABCC3 expression in glioma tissue and normal brain tissues; C: ABCC3 expression levels in different subtypes of glioma)

The expression level of the *ABCC3* gene was different in various solid tumors (Figure. 1A). The expression level of the *ABCC3* gene in glioma tissues was lower than that of normal brain tissues, Figure. 1B; In different subtypes of glioma, there was no significant difference in the expression level of *ABCC3* between tumors and normal brain tissues (Figure. 1C). This may partly due to the small sample size of the subgroups with lower statistical power.

### 3.2 PPI web and Hubb gene analysis

**Figure 2:**
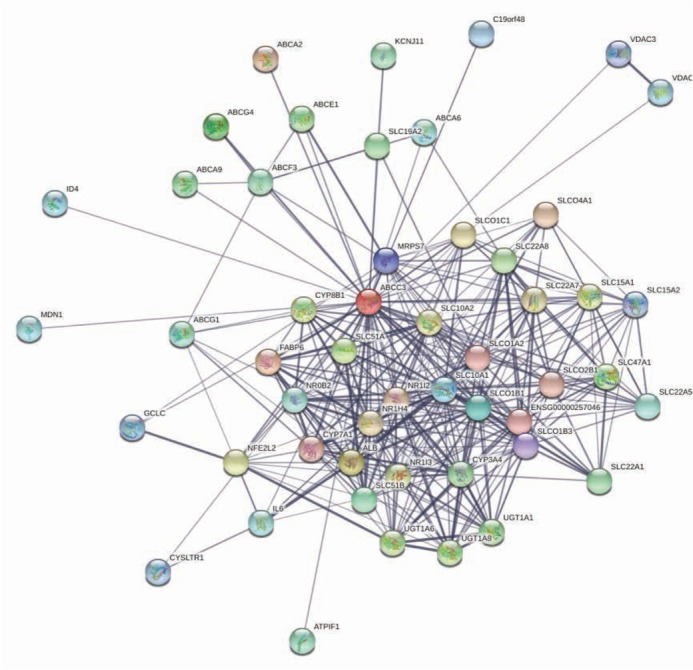
PPI Network for ABCC3 and related proteins

**Figure 3:**
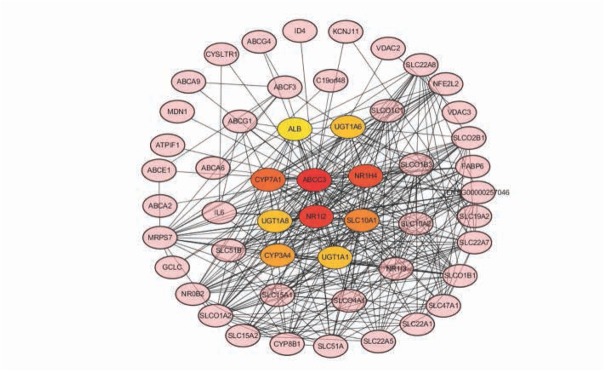
Network of hub genes identification by cytoscape

The PPI network contains 51 nodes with the average node degree of 13.3 and the local clustering coefficient of 0.72 which indicated that the PPI enrichment was significant (p<0.001), Figure 2. Ten hub genes (*ABCC3, NR1I2, NR1H4, CYP7A1, SLC10A1, CYP3A4, UGT1A1, UGT1A8, UGT1A6 and ALB*) were identified by the cytoscape software, Figure 3.

### 3.3 GO and KEGG enrichment of *ABCC3* and related hub genes

For cellular component category, the *ABCC3* and hub genes were enriched in basolateral plasma membrane, endoplasmic reticulum chaperone complex, integral component of membrane and et c. For the biological process, the genes were enriched in negative regulation of cellular glucuronidation, negative regulation of cellular glucuronidation, drug export and et c process. And in the aspect of molecular function, it was enriched in active transmembrane transporter activity, organic hydroxy compound transmembrane transporter activity, lipid-transporting ATPase activity and et c. The KEGG pathway was enriched in drug metabolism - cytochrome P450 and PPAR signaling pathway.

### 3.4 Expression Correlation analysis of *ABCC3* in Glioma

**Figure 4:**
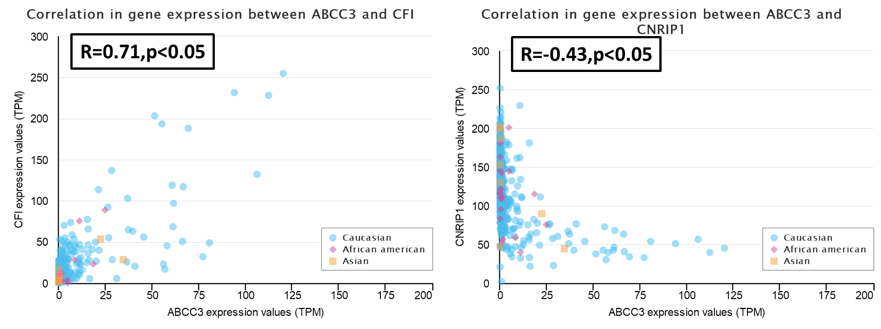
Positive and negative expressioncorrelation analysis of ABCC3 in glioma(A: positive correlation of CFI gene with ABCC3; B:negative correlation of CNRIP1 gene with ABCC3)

We selected the top two genes (*CFI* and *CNRIP1*) that most positively or negatively correlated with *ABCC3* to analysis. The *CFI* gene expression level was positively correlated with the *ABCC3* expression level (r=0.71, p<0.05). And the *CNRIP1* gene expressed was negative correlated with *ABCC3* expression (r=-0.43, p<0.05), Figure 4.

### 3.5 Survival analysis of *ABCC3*

**Figure 5:**
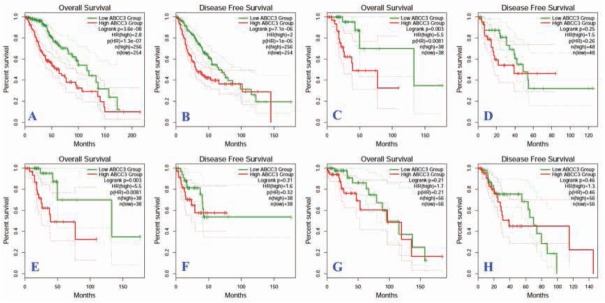
Survival curves of prognostic relationship between ABCC3 high and low expression group in glioma patients (A: comparison of overall survival of glioma patients; B: comparison of progression-free survival of glioma patients; C: comparison of overall survival of astrocytoma; D: comparison of progression-free survival of astrocytoma; E: comparison of overall survival of oligoastrocytoma; F:comparison of progression-free survival of oligoastrocytoma; G: Total survival comparison of oligodendroglioma; G:progression-free survival comparison of oligodendroglioma)

According to the median expression level of *ABCC3*, the patients were divided into high and low expression groups. A log-rank test was used to compare the survival time of high and low expression groups. The overall survival (HR=2.8, P<0.05) and disease-free survival rates (HR=2.0, P<0.05) of patients with *ABCC3* low expression glioma were significantly higher than those of patients with high expression of ABCC3, Figure. 5A, B. Subgroup analysis showed that the overall survival of patients with astrocytoma (Figure.5C) and oligoastrocytoma (Figure. 5E) with high expression of *ABCC3* was significantly lower than that of patients with low expression of *ABCC3* (P<0.05), but there was no significant difference in disease progression survival (P> 0.05). (Figure. 5D, F, G, H.)

### 3.6 Pan cancer analysis of the hub genes

**Figure 6:**
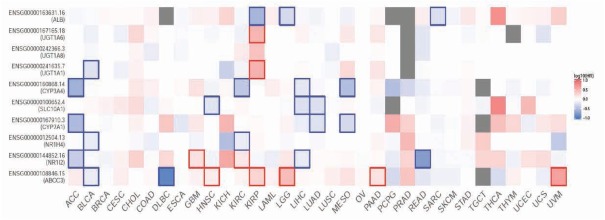
Survival map of hazard ratio(HR) of ABCC3 and hub genes for pan cancer analysis

The hazard ratio (HR) of survival for *ABCC3* and hub genes were demonstrated in Figure 6. High expression of ABCC3 was correlative with poor prognosis of head and neck squamous carcinoma (HNSC), kidney renal clear cell carcinoma (KIPR), brain lower grade glioma (LGG), pancreatic adenocarcinoma (PAAD) and uveal melanoma(UVM).

## 4 Discussion

Glioma is one of the most diagnosed carcinoma of the human central nervous system. The most malignant intracranial tumors are grade IV glioblastoma (GBM). In Europe and the U.S, the incidence of the disease among the general population is 3-5/100,000 [13]. GBM may develop from a low-grade glioma (secondary GBM) with poor prognosis. Even with the best surgical excision combined with chemotherapy and radiotherapy, the average survival of GBM is about 12 to 15 months [14, 15]. However, the exact pathogenesis of glioma is still unclear [16, 17]. In recent years, with the development of molecular biology technology, more and more evidence has shown that the occurrence and prognosis of glioma is related to the different expressed oncogenes and tumor suppressor genes [18, 19, 20].

The protein encoded by *ABCC3* is a member of the superfamily of ATP-binding cassette (ABC) transporters. The specific function of this protein has not yet been determined. According to the preset studies, protein coded by the *ABCC3* gene may play a role in the transport of biliary and intestinal excretion of organic anions. However, its biological function in carcinoma, especially in glioma, was unclear [21].

In the present work, we used bioinformatics analysis to investigate the expression of the *ABCC3* gene in glioma, its related signaling pathway and its relationship with prognosis of glioma patients. The results suggested that the expression of *ABCC3* in glioma tissues was significantly lower than that of normal brain tissues, suggesting that *ABCC3* may play an important role in the occurrence and development of glioma. The decreased expression of *ABCC3* may be due to the promoter hypermethylation or histone modification of the ABCC3 gene. The KEGG was enriched in drug metabolism - cytochrome P450 response to xenobiotic stimulus and PPAR signaling pathway. However, the known pathway of *ABCC3* may not correlate with the development of glioma. We also found that the general OS and DFS of patients with *ABCC3* overexpression in all kinds of glioma were significantly lower than those of patients with low expression of *ABCC3* which indicated that high expression of *ABCC3* may act as a poor molecular marker for glioma patients prognosis. However, the subgroup analysis according to the glioma pathology type showed that the overall survival of patients with astrocytoma and oligoastrocytoma with high expression of *ABCC3* was significantly lower than that of patients with high expression of *ABCC3* (P<0.05), but there was no significant difference in disease progression survival (P> 0.05). The non-signification for disease progression survival may be due to the small sample size in the evaluation of the disease progression survival with low statistical power.

However, this work had had limitation in that the results need further validation by experiment in vitro and in vivo. And the prognostic value of ABCC3 also needs verification by clinical trials.

## 5 Conclusion

The expression level of *ABCC3* gene in glioma was low compared to normal brain tissue. The overall survival and disease-free survival of in *ABCC3* low-expression group was significantly decreased. This finding indicated that the *ABCC3* gene may play an important role in the development of human glioma and could be used as biomarker for prognosis.
